# Focus on Cellular Iron Metabolism in Aortic Disease

**DOI:** 10.31083/j.rcm2305169

**Published:** 2022-05-11

**Authors:** Yoshiro Naito, Masaharu Ishihara

**Affiliations:** ^1^Department of Cardiovascular and Renal Medicine, Hyogo Medical University, 663-8501 Nishinomiya, Japan

**Keywords:** abdominal aortic aneurysm, atherosclerosis, hepcidin, iron, transferrin receptor 1

## Abstract

**Background:**

Iron deficiency leads to health problems. Conversely, iron overload induces the generation of reactive oxygen species and health problems. Body iron status contributes to the development of various diseases, including aortic disease. Indeed, several clinical studies have reported that iron status can be linked to the pathogenesis of aortic disease. At the cellular level, iron uptake is regulated by the cellular iron transporter, transferrin receptor 1, while systemic iron homeostasis is regulated by hepcidin. As body iron status is regulated to maintain cellular and systemic iron homeostasis, iron metabolism in aortic disease is puzzling and not well understood.

**Methods:**

Perspective and short communication.

**Conclusions:**

This review provides an overview of the relevant research investigating the association between cellular iron metabolism and aortic disease.

## 1. Introduction

Iron deficiency contributes to health problems such as anemia [1]. On the other hand, iron overload induces the generation of reactive oxygen species via the Fenton reaction (${\mathrm{Fe}}^{2+}$ + $H_{2}$$O_{2}$ → ${\mathrm{Fe}}^{3+}$ + •OH + ${\mathrm{OH}}^{-}$) and can cause cell damage and harmful effects on the body. Therefore, iron overload is also associated with the pathogenesis of various health problems [2, 3]. Body iron status is regulated to maintain cellular and systemic iron homeostasis. Iron binds to transferrin in the blood, but if surplus iron transcends the carrying capacity of transferrin, non-transferrin-bound iron circulates and causes tissue injury, including aortic disease. Indeed, various clinical studies have shown that iron status is associated with the pathogenesis of aortic disease [4, 5, 6, 7, 8, 9, 10, 11]. Iron metabolism in aortic disease is puzzling, and the association between cellular iron metabolism and aortic disease remains obscure. This short communication investigates the relation between cellular iron metabolism and aortic disease.

## 2. Clinical Investigation on Iron and Aortic Disease

**Fig. 1.**
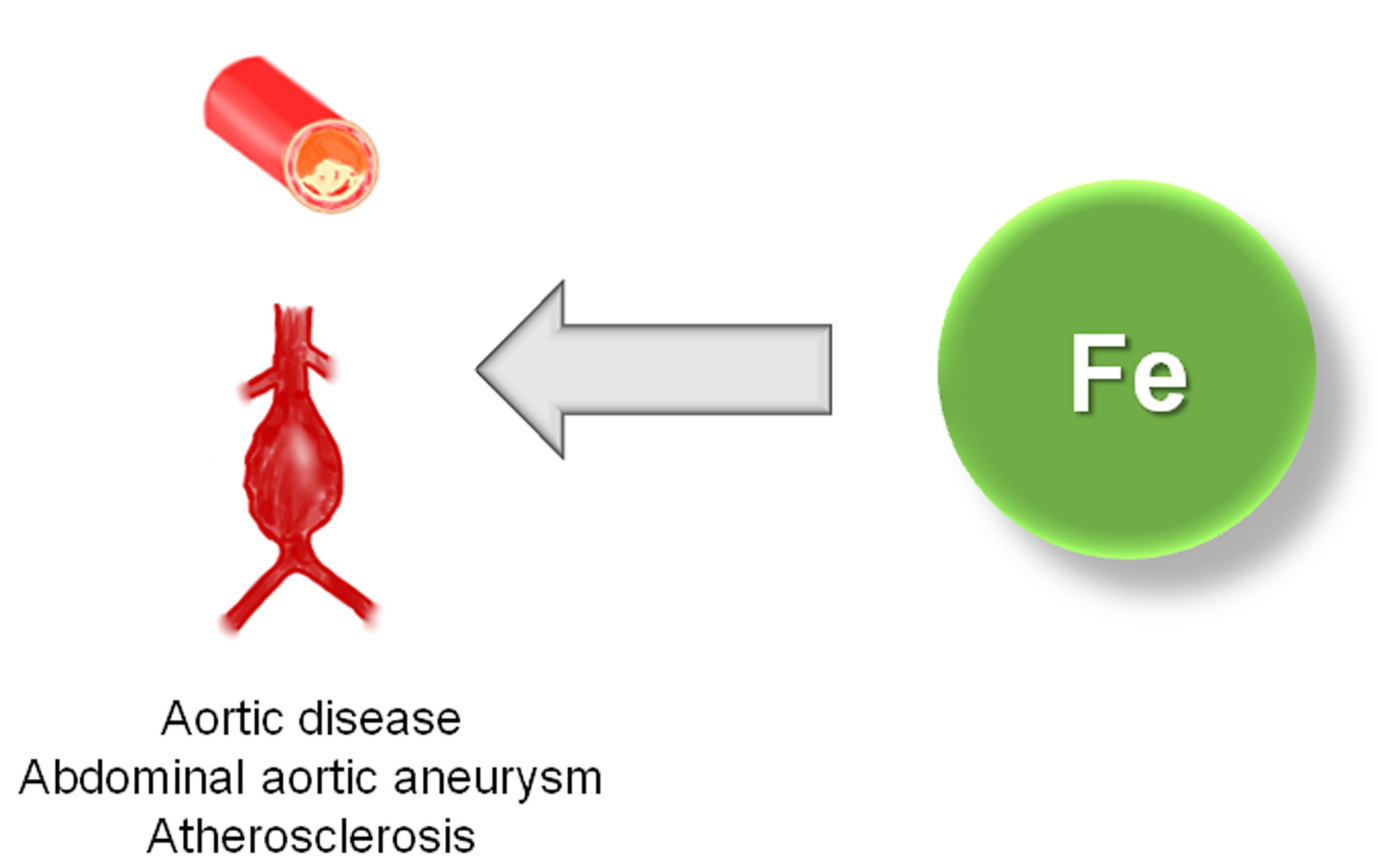
**Iron is associated with the pathogenesis of aortic disease**. Iron is associated with the pathogenesis of aortic disease, such as abdominal aortic aneurysm and atherosclerosis. In addition to systemic iron levels, local iron levels and iron metabolism in the aortic region should be considered.

Clinical studies have proposed iron status is harmful or beneficial for aortic disease (Table 1, Ref. [4, 5, 6, 7, 8, 10, 11]). A number of clinical studies have reported an association between iron overload and aortic disease [4, 5, 6, 7, 8, 9]. Most of these reports have used serum ferritin levels as a marker of iron levels [4, 5, 6]. In contrast to these reports, a previous study has reported that higher iron status lowers the risk of coronary artery disease [10]. The Ludwigshafen Risk and Cardiovascular Health Study showed that iron depletion is associated with coronary artery disease [11]. Taken together, these findings indicate that iron is associated with the pathogenesis of aortic disease (Fig. 1). However, optimal systemic iron levels in aortic disease are still controversial. When understanding the pathogenesis of aortic disease, it is important to consider cellular iron levels and iron metabolism in the aortic region, in addition to systemic iron levels.

**Table 1.** **Clinical investigation on iron and aortic disease**.

Harmful effects of iron on aortic disease	Beneficial effects of iron on aortic disease
An association between high stored iron levels and a risk of myocardial infarction [4]	An association between increased body iron and reduced coronary artery disease risk [10]
An association between body iron stores and the progression of carotid atherosclerosis [5]	An association between iron depletion and coronary artery disease [11]
An association between increased serum ferritin levels and the presence of coronary artery calcium score [6]	
An association between iron chelation and endothelial function in patients with coronary artery disease [7]	
An association between serum ferritin levels and peripheral arterial disease [8]	

## 3. Transferrin Receptor 1 in Abdominal Aortic Aneurysm

Although there have been several clinical studies on iron and aortic disease, the role of cellular iron metabolism in the pathogenesis of aortic disease remains largely unknown. Cellular iron uptake is regulated by the cellular iron transporter, transferrin receptor 1 (TfR1) [12]. TfR1 is ubiquitously expressed in each tissue and has been shown to play a role in several diseases.

Regarding aortic disease, we have shown the role of TfR1 in the pathogenesis of abdominal aortic aneurysm (AAA). First, we found iron deposition in human AAA walls by Berlin blue staining. In addition, we found that the Berlin blue-positive area was similar to the positive areas of a macrophage marker, CD68 and the oxidative stress marker, ${\mathrm{8-Hydroxy-2}}^{\prime }$-eoxyguanosine, indicating that iron plays a role in the pathogenesis of AAA and is related to inflammation and oxidative stress in human AAA [13]. Moreover, we found that aortic iron accumulation was observed in angiotensin II-induced AAA mice with increased inflammation and oxidative stress. In addition, iron restricted diet inhibited the development of AAA by attenuating aortic inflammation and oxidative stress in these mice [13].

**Fig. 2.**
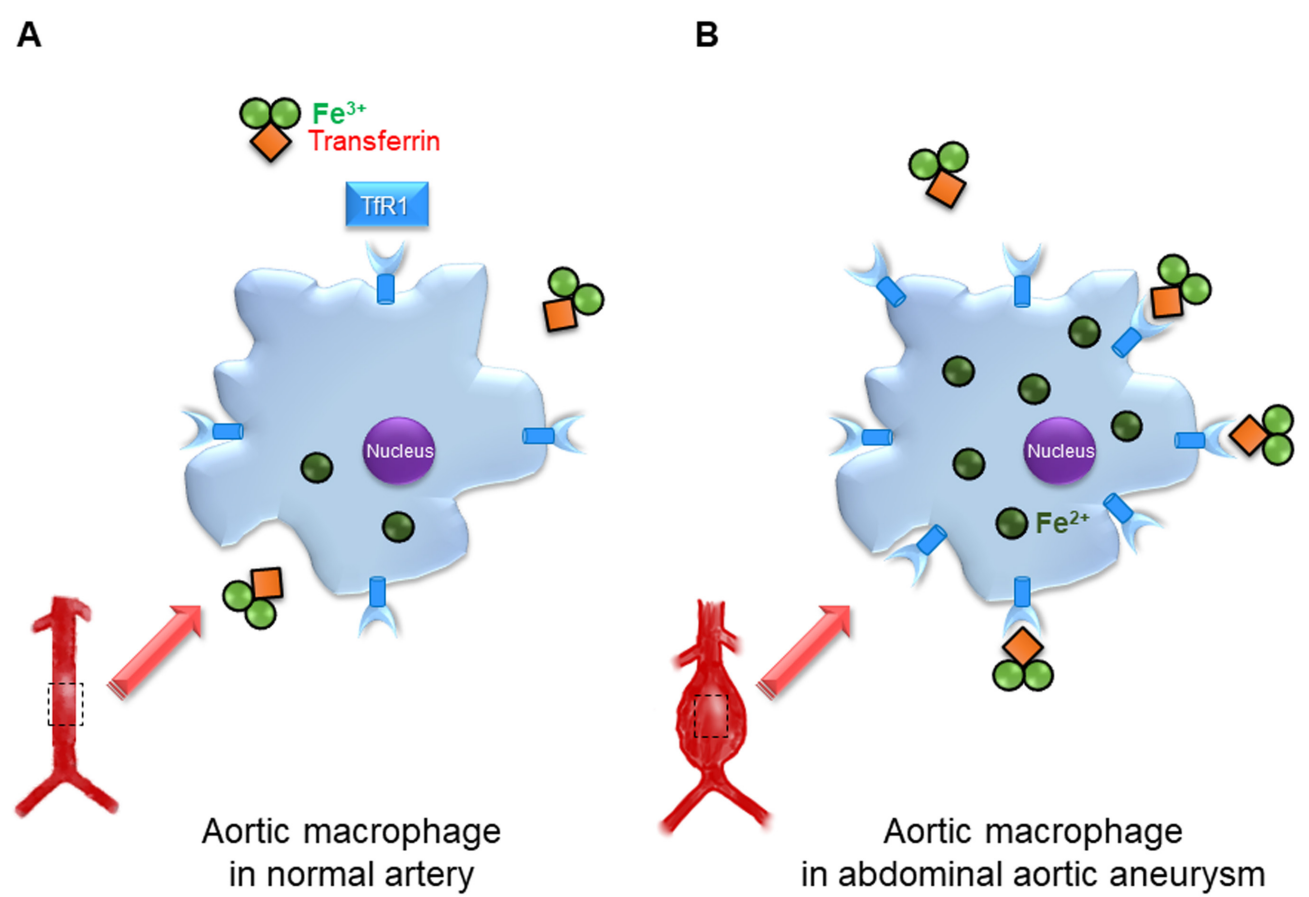
**Transferrin receptor 1 in the pathogenesis of abdominal aortic aneurysm**. (A) Transferrin receptor 1 (TfR1) is expressed in aortic macrophages of normal artery. (B) In abdominal aortic aneurysm (AAA) walls, increased TfR1 expression in aortic macrophages induces intracellular iron accumulation, and this may promote AAA progression.

In these studies, immunohistochemical analysis demonstrated that TfR1 was expressed in AAA walls. Of note, increased aortic TfR1 expression was observed in murine and human AAA walls, and the TfR1 positive area was consistent to areas where a macrophage marker, F4/80, and iron accumulation occurred in murine AAA walls. TfR1 is upregulated under cellular iron-deficient conditions and accelerates cellular iron uptake. Conversely, TfR1 is downregulated under cellular iron overload conditions and inhibits cellular iron uptake [12]. Since increased iron deposition and TfR1 expression were observed in murine and human AAA walls, these results indicate that dysregulated TfR1 may contribute to aortic iron overload in AAA walls (Fig. 2). As regard to TfR1 in aortic disease, we have also shown increased aortic TfR1 expression in hypertensive model rats [14, 15]. Taken together, these results suggest the involvement of aortic TfR1 in the pathogenesis of aortic disease.

## 4. Hepcidin in Aortic Disease

Systemic iron metabolism is regulated by hepatic hormone hepcidin and is kept by duodenal iron absorption. Hepcidin is upregulated in response to iron overload or inflammation, and is downregulated by iron deficiency [16]. Dietary reducted ferrous iron is exported to the blood by the iron transporter, ferroportin [17].

Hepcidin is reported to be a master iron regulator in the pathogenesis of aortic disease. The Nijmegen Biomedical Study showed that serum hepcidin levels were associated with the presence of aortic plaques in postmenopausal women in the general population. This study also reported that the hepcidin/ferritin ratio was associated with the ankle-brachial index in men and women [18].

**Fig. 3.**
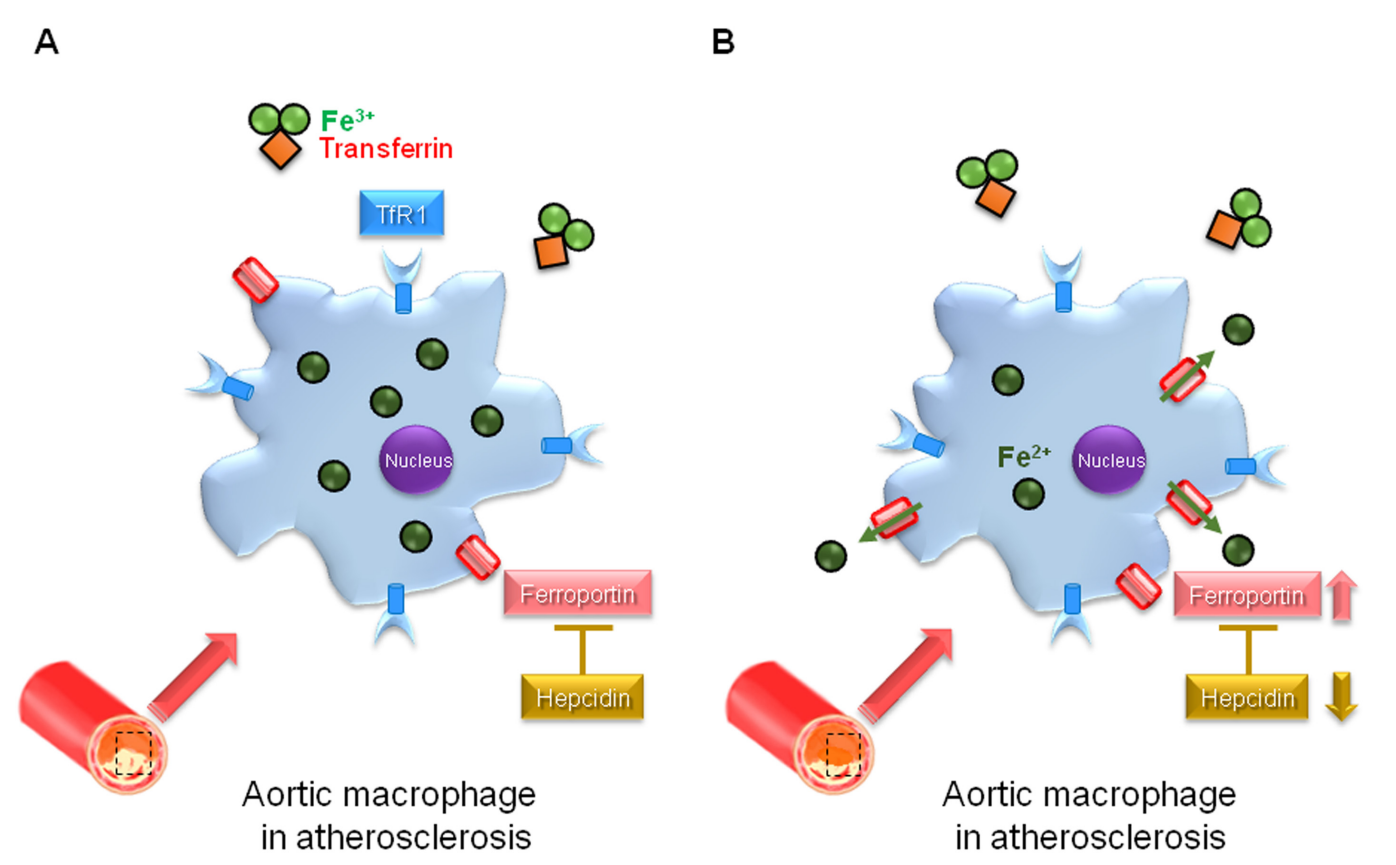
**Hepcidin in the pathogenesis of atherosclerosis**. (A) In atherosclerotic lesion, hepcidin suppresses cellular iron export from aortic macrophages with downregulation of ferroportin expression and enhances intracellular iron accumulation, and this may promote atherosclerosis. (B) In hepcidin gene-deficient mice with low-density lipoprotein receptor deficient mice, cellular iron export is induced from aortic macrophages with upregulation of ferroportin expression and decreases intracellular iron accumulation, this may suppress atherosclerosis.

Hepcidin regulates ferroportin expression [17]. Hepcidin binds to ferroportin, inducing its degradation, then inhibiting cellular iron export from duodenocytes, hepatocytes, and macrophages. If hepcidin is absent, ferroportin in the cell membrane results in the export of cellular iron from these cells. Malhotra *et al*. [19] reported the role of hepcidin in aortic disease using hepcidin gene-deficient mice. Crossing hepcidin gene-deficient mice with low-density lipoprotein receptor-deficient mice was associated with a decrease in atherosclerotic lesions compared to control mice. These mice showed reduced atherosclerosis with reduced aortic macrophage iron and macrophage proinflammatory phenotype, compared to control mice after a high-fat diet. Hepcidin inhibits cellular iron export from macrophages by downregulating ferroportin expression and enhancing intracellular iron accumulation. Intracellular iron accumulation leads to the composition of oxidized low-density lipoprotein cholesterol and proinflammatory intermediates and intracellular reactive oxygen species generation, which may promote atherosclerotic plaque progression. Collectively, these results suggest that decreased aortic macrophage proinflammatory phenotypes might contribute to reduced atherosclerosis with decreased hepcidin levels (Fig. 3). However, it is unknown whether decreased hepcidin levels could affect aortic disease in humans.

## 5. Focus on Cellular Iron Metabolism in Aortic Disease

Similar to our AAA study, several experimental studies have shown iron accumulation in atherosclerotic lesions [20, 21]. For instance, iron accumulation was reported in the endothelium, smooth muscle cells, and intima enriched foam cells in the aorta of apolipoprotein E-deficient mice [20]. In addition, hereditary hemochromatosis mice crossed with apolipoprotein E-deficient mice showed iron deposition in the media layer of the aorta and enhanced atherosclerotic lesions [21]. Collectively, an investigation into cellular iron metabolism could reveal the pathogenesis of aortic disease such as AAA and atherosclerosis, and the causal link between iron and aortic disease.

## 6. Conclusions

Iron is associated with the pathogenesis of aortic disease, such as AAA and atherocclerosis. The investigation of cellular iron metabolism could help to clarifying the complex association between iron and aortic disease. Although further studies are necessary, a focus on cellular iron metabolism in aortic disease may result in new therapeutic strategies for the condition.

## Abbreviations

AAA, abdominal aortic aneurysm; TfR1, transferrin receptor 1.
